# Synthesis of Novel 3-Aryl-*N*-Methyl-1,2,5,6-Tetrahydropyridine Derivatives by Suzuki coupling: As Acetyl Cholinesterase Inhibitors

**DOI:** 10.2174/1874104500701010004

**Published:** 2007-09-05

**Authors:** S.B. Benaka Prasad, Y.C. Sunil Kumar, C.S. Ananda Kumar, C.T Sadashiva, K Vinaya, K.S Rangappa

**Affiliations:** Department of Studies in Chemistry, University of Mysore, Manasagangotri, Mysore, Mysore-570 006, India

**Keywords:** Tetrahydropyridine, Suzuki coupling, AChE, Alzheimer's disease.

## Abstract

Alzheimer’s disease (AD) is a neurodegenerative disorder affecting the central nervous system, which is also associated with progressive loss of memory and cognition. The development of numerous structural classes of compounds with different pharmacological profile could be an evolving, promising therapeutic approach for the treatment of AD. Thus, providing a symptomatic treatment for this disease are cholinomimetics with the pharmacological profile of Acetylcholinesterase (AChE) inhibitors. In view of this, we have synthesized novel 3-aryl-*N*-methyl-1,2,5,6-tetrahydropyridine derivatives **5a-k** by Suzuki coupling and screened the efficacy of these derivatives for their AChE inhibitor activity.

## 1 INTRODUCTION

Alzheimer's disease (AD) is an irreversible, progressive neurodegenerative disorder that occurs gradually and results in memory loss, unusual behavior, personality changes and a decline in thinking abilities [1]. Taking into account the increase in life expectancy, the fact that the incidence of AD increases with advancing age and the devastating effects of this illness, nowadays AD represents a major public health problem and will presumably be the most important pathology of the 21^st^ century in the developed countries. Efforts have been made in the last two decades in order to determine the etiopathogenesis of AD and to carry out its early diagnosis and therapeutic control [2, 3]. Survival disease is variable in patients with AD and they usually die of infections, with death occurring approximately 10 years after the onset of symptoms. Thus, AD is the third largest cause of death in the western world after cardiovascular diseases and cancer. As a consequence, potentiation of central cholinergic action has become an effective approach for the palliative treatment of mild to moderate cases of AD [4]. Several therapeutic strategies have been explored to enhance cholinergic neurotransmission in order to alleviate some of the symptoms of AD. These include the use of Acetylcholinesterase inhibitors, the administration of acetylcholine precursors, the investigation of acetylcholine releasers and direct acetylcholine receptor agonists [5]. Among these strategies, inhibition of AChE has proven to be the most successful means to balance the cholinergic deficit and to stabilize the symptoms [6]. Two main strategies can be adapted in the treatment of AD, alleviating the cognitive deficits and slowing down degenerative process. Current therapy comes under the first approach [7]. To date, the cholinesterase inhibitors have been shown to improve some aspects of cognitive performance, however, efficacy has been poor and side effects are problematic [8-10]. A variety of compounds particularly cholinesterase inhibitors, were used in an attempt to increase cholinergic activity [11, 12]. Physo-stigmine (1,2,3,3a,8,8a-hexahydro-1, 3a,8-trimethyl-,methyl-carbamate(ester),(3aS,8aR)-pyrrolo(2,3- b)indol-5-ol), was one of the earlier acetylcholinesterase (AChE) inhibitors studied, produced modest improvements in cognition, but its use was limited because of frequent dosing regimen and severe adverse reactions [13-15]. The first available treatment for AD was THA (1,2,3,4-tetrahydro-9-aminoacridine), which demonstrated moderate but significant efficacy [16]. Arecoline (Methyl 1,2,5,6-tetra-hydro-1-methylpyridine-3-carboxylate) is a naturally occurring alkaloid with cholinergic properties as a tertiary amine. This muscarinic agonist has strong lipophilic properties and can easily cross the blood-brain barrier after systemic administration [17]. Many experimental studies in animals [18, 19] as well as few clinical studies in demented patients and normal volunteers [20, 21] have demonstrated the ability of arecoline to prevent or to reduce memory deficit. Due to rapid *in vivo* hydrolysis of the ester functional group, arecoline has an extremely short half life and negligible activity after oral administration [22, 23]. Based on the knowledge acquired from the basic studies of several known arecoline derivatives about the structural requirements for potent AChE inhibitors, a series of *N*-methyl tetrahydropyridine-3 derivatives were been synthesized and screened for their efficacy as AChE inhibitors.

## 2 RESULTS AND DISCUSSION

### 2.1 Chemistry

**Scheme 1:**
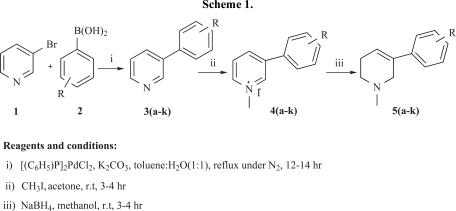


The preparation of novel 3-aryl-*N*-methyl-1,2,5,6-tetrahydropyridine derivatives **5a-k** is summarized in Scheme **1**, *via* Suzuki coupling by using commercially available 3-bromopyridine as starting material. The synthesis involves three steps; the first step involves the Suzuki coupling, where mixture of 3-bromopyridine and substituted aryl boronic acids in toluene: H_2_O (1: 1) was refluxed overnight using bis(triphenylphosphine) palladium (II) dichloride as a catalyst in the presence of the mild base K_2_CO_3_. The presence of aromatic proton peaks at 6.9-7.8 ppm in the ^1^H NMR spectra confirms the formation 3-aryl pyridines **3a-k**. The second step is the *N*-methylation using Methyl iodide in acetone as a solvent at room temperature and the formation of the derivatives **4a-k** was confirmed by the presence of singlet methyl peak at 2.51 ppm. The third step is the reduction of the *N-*substituted pyridine **5a-k** using NaBH_4_ in Methanol, the presence of a triplet of an olefinic proton at 6.2-6.35 ppm and also the multiplets for aliphatic proton at 2.4-3.3 ppm in the ^1^H NMR spectra, confirming the partial reduction of the pyridine ring. All the synthesized compounds **5a-k** obtained in good yield ranged from 77-92% with high purity. The chemical structures, physical data and purity of the derivatives are shown in Table 1.

### 2.2 AChE activity

The inhibitory activities of the novel synthesized compounds against AChE were studied using the method of Ellman *et al.* [24]. Determination of the rate of hydrolysis of Acetylthiocholine (ATCh) in the presence of inhibitors against different sources such as electric eel AChE, human serum AChE and rat brain homogenate AChE is shown in Figs. (**1-3**), respectively. The comparative inhibitory activities of **5a-k** are tabulated in Table 2.

Activities of the synthesized compounds were compared with the inhibitory activity shown by the known standard inhibitor Neostigmine [25]. Activity was 50% at a dose of **50**.**2**, **63**.**4**, **122**.**0** and **176**.**8 nM** for compounds **5a**, **5b**, **5h** and **5k** respectively. Compounds **5d**, **5f**, **5g** and **5j** showed less inhibitory activity while compounds **5c**, **5e** and **5i** showed no inhibition. The pharmacological conclusion was that, trifluoro methyl phenyl ring in compound **5a** (**IC_50_** =**49**.**5**, **52**.**1**, **50**.**2**) effectively blocked the enzyme compared to the rest of the derivatives studied. The 4-Cyano phenyl ring in **5b** (**IC_50_** =**64**.**0**, **68**.**0**, **63**.**6**), 2-fluoro-4-biphenyl ring in **5h** (**IC_50_** =**118**.**0**, **115**.**6**, **122**.**6**) and 2-fluoro-3-chlorophenyl ring in **5k** (**IC_50_** =**182**.**3**, **170**.**0**, **176**.**8**) derivatives were also effective in blocking the AChE enzyme activity of electric eel, human serum and rat brain homogenate, respectively, which led to the suggestion that compound **5a** was considered as a potent AChE inhibitor.

Among the synthesized molecules, compounds **5a** and **5b** showed good AChE inhibition when compared to the other molecules with Neostigmine. This is probably due to the presence of electron withdrawing group trifluoromethyl (-CF_3_) in **5a** and cyano (-CN) in **5b**. From the results obtained, it was revealed that the electron withdrawing substituent on phenyl ring increases the inhibition, as observed from the derivatives **5a**, **5b**, **5k**, **5h** and **5f**. The presence of electron donating groups on phenyl ring (**5e**, **5i** and **5j**) had no AChE inhibition. The aromatic fluoro substituent is slightly smaller than hydrogen in terms of molecular refractivity (MR) being 0.09 versus 0.10. Therefore, the fluoro substituent acts as a potent inhibitor. The molecules synthesized herein were expected to enter the central nervous system (CNS) because of the structural similarity with Arecoline. The smaller the substituent, it raised greater expectations of a more favorable activity at tetrahydropyridine-3 site of the molecule. From the SAR, it was observed that the *N*-methyl tetrahydropyridine-3 derivatives with trifluoromethyl (**5a**) and cyano substituents (**5b**) showed better activity than the other derivatives mentioned. The results of reversing amnesic effect of scopolamine induced memory loss in passive avoidance step-down task paradigm in rat [24] are shown in Table 3. Compound **5a**, **5b** and **5h** minimized the number of mistakes done to 13, 15 and 18 from 34 mistakes done by scopolamine induced memory loss. Our findings revealed that compounds **5a**, **5b** and **5h** improved scopolamine induced impairment of memory registration in passive avoidance behavior in mice. It is well known that the cholinergic system plays an important role in learning and memory [26] and there is evidence from both animal and human studies indicating that learning and memory can be modified by drugs that affect central cholinergic function [27]. In light of these findings, our results suggest that the antiamnesic effect of **5a**, **5b** and **5h** on scopolamine-induced impairment of learning and memory may be related to modification of cholinergic neuronal systems. The results of compounds showed that *in vivo* and *in vitro* results are comparable. It can be concluded from this study that for effective binding and blocking of the AChE activity, the molecule needs to bind with peripheral and active site of the enzyme and some *N*-methyl tetrahydropyridine-3 derivatives with substituents such as fluoro, cyano or biphenyl that may bind to the active site. The substituents like Cl, OH, OCH_3_ and OC_2_H_5_ containing aromatic rings bind to peripheral site of the enzyme. The structures of potent AChE inhibitors are shown in Fig. (**4**).

## 3 CONCLUSION

Derivatives of *N*-methyl tetrahydropyridine-3 having different substituents in the aryl residue were tested for their activity for the substrate acetylcholine iodide. The order of potency is **5a>5b>5h>5k**. The other compounds screened failed to elicit any inhibition of acetyl cholinesterase from electric eel, human serum rat brain homogenate. The molecules synthesized herein can be expected to enter the central nervous system (CNS) because of the structural similarity with Arecoline. Two of the analogs synthesized **5a** and **5b** have AChE inhibitory activity and may provide new leads in the search for effective AChE inhibitory agents. Therefore, it has been summarized that substituting heterocyclic ring to the aromatic ring on *N*-methyl tetrahydropyridine-3 derivatives may increase the efficacy of AChE inhibitory activity and to achieve this, the work is under progress.

## 4 EXPERIMENTAL

Infrared (IR) spectra were recorded using a Jasco FTIR-4100 series. Nuclear magnetic resonance (^1^H-NMR) spectra were recorded on Shimadzu AMX 400-Bruker, 400MHz spectrometer using D_6_ DMSO as a solvent and TMS as an internal standard (chemical shift in δ ppm). Mass and purity were recorded on a LC- MSD-Trap-XCT. Elemental (CHNS) analysis was obtained on Vario EL III Elemental Analyzer. Silica gel column chromatography was performed using Merck 7734 silica gel (60-120 mesh) and Merck made TLC plates.

### 4.1 General Procedures for the Synthesis of 3-Aryl pyridines 3a-k

To a solution of 3-bromopyridine (1.0 equiv) in toluene and H_2_O (1: 1), K_2_CO_3_ (3.0 equiv) was added and stirred for 10 min at room temperature under N_2_, followed by the addition of (Ph_3_P)_2_PdCl_2_ (0.1 equiv) and substituted aryl boronic acids (1.1 equiv). The mixture was refluxed overnight (JinHeng Tetrahedron Lett 2006) [28]. The progress of the reaction was monitored by TLC. After completion of the reaction, the mixture was filtered through celite to remove the palladium particles and the filtrate was evaporated under reduced pressure. The residue was taken in H_2_O, extracted with Ethyl acetate and dried over anhydrous Na_2_SO_4_. Pure compounds been obtained by column chromatography using Hexane/Ethyl acetate (8: 2) as an eluent and the compounds were characterized by ^1^H-NMR spectroscopy and LCMS.

### 4.2 General Procedures for the Synthesis of *N*-methylpyridine-3 Derivatives 4a-k

The compounds **3a-k** were taken in the 10-fold volume of acetone, then methyl iodide (4.0 equiv) was added and the mixture stirred for 3-4 hr. After completion of the reaction, the reaction was monitored by TLC and the solvent was evaporated under reduced pressure. A yellow pyridinum salt **4a-k** was obtained and taken for reduction [29].

### 4.3 General Procedures for the Synthesis 3-aryl-*N*-methyl-1,2,5,6-tetrahydropyridine Derivatives 5a-k

The compounds **4a-k** were taken in MeOH, NaBH_4_ (3.0 equiv) was added and the mixture stirred at rt for 3-4 hr. After completion of the reaction, the reaction was monitored by TLC. H_2_O was added and extracted with Ethyl acetate. Pure compounds were obtained as oils after evaporation of the solvent under reduced pressure. All the compounds were characterized by ^1^H-NMR, LC/MS, FTIR and CHN analysis.

#### 4.3.1 Synthesis of 3-[4-(trifluoromethyl)phenyl]-1,2,5,6-tetrahydro-1-methylpyridine 5a

Obtained from 1-methyl-3-(4-trifluoromethyl-phenyl) pyridinium salt (**4a**), (0.5 g, 1.36 mmol), NaBH_4_ (0.257 g, 6.80 mmol). Oily liquid (0.278 g, 85%). ^1^H-NMR (CDCl_3_, 400MHz) δ: 7.74 (d, 2H, Ar-H), 7.45 (d, 2H, Ar-H), 6.35 (t, 1H, -CH), 3.33 (s, 2H, -CH), 2.62 (t, 2H, -CH), 2.50 (s, 3H, -CH), 2.41 (m, 2H, -CH_2_). IR (KBr, cm^-1^): 3045, 2931, 2615, 1616, 1465, 1173. MS (ESI) *m/z*: 242.15(M+H^+^). Anal. calc.for C_13_H_14_F_3_N (in %): C-64.72, H-5.85, N-5.81. Found C-64.68, H-5.81, N-5.78.

#### 4.3.2 Synthesis of 3-(4-cyanophenyl)-1,2,5,6-tetrahydro-1-methylpyridine 5b

Obtained from 3-(4-cyano-phenyl)-1-methyl-pyridinum salt (**4b**), (0.5 g, 1.55 mmol), NaBH_4_ (0.293 g, 7.75 mmol). The product obtained was oily liquid (0.245 g, 80%). ^1^H-NMR (CDCl_3_, 400MHz) δ: 7.72(d, 2H, Ar-H) 7.49 (d, 2H, Ar-H), 6.35 (t, 1H, -CH), 3.33 (s, 2H, -CH), 2.62 (t, 2H, -CH), 2.53 (s, 3H, -CH), 2.41 (m, 2H, -CH_2_). IR (KBr, cm^-1^): 3025, 2945, 2630, 2223, 1630. MS (ESI) *m/z*: 199.10(M+H^+^). Anal. Calc. for C_13_H_14_N_2_ (in %): C-78.75, H-7.12, N-14.13. Found C-78.72, H-7.09, N-14.00.

#### 4.3.3 Synthesis of 3-(3-acetylphenyl)-1,2,5,6-tetrahydro-1-methylpyridin 5c

Obtained from 3-(3-acetyl-phenyl)-1-methyl-pyridinium salt (**4c**), (0.5 g, 1.07 mmol), NaBH_4_ (0.202 g, 5.36 mmol). The product obtained was oily liquid (0.178 g, 77%). ^1^H-NMR (CDCl_3_, 400MHz) δ: 7.4 (s, 1H, Ar-H), 7.42 (d, 1H, Ar-H), 7.22 (m, 2H, Ar-H) 6.34 (t, 1H, -CH), 3.33 (s, 2H, -CH), 2.62 (t, 2H, -CH), 2.60 (s, 3H, -COCH_3_), 2.52 (s, 3H, -CH), 2.41 (m, 2H, -CH_2_). IR (KBr, cm^-1^): 3028, 2940, 2618, 1670, 1628. MS (ESI) *m/z*: 217.26(M+H^+^). Anal. Calc. for C_14_H_17_NO (in %): C-78.10, H-7.96, N-6.51. Found C-78.06, H-7.92, N-6.48.

#### 4.3.4 Synthesis of 3-(4-chlorophenyl)-1,2,5,6-tetrahydro-1-methylpyridine 5d

Obtained from 3-(4-chloro-phenyl)-1-methyl-pyridinium salt (**4d**), (0.5 g, 1.51 mmol), NaBH_4_ (0.28 g, 7.55 mmol). The product obtained was oily liquid (0.275 g, 88%). ^1^H-NMR (CDCl_3_, 400MHz) δ: 7.32 (d, 2H, Ar-H) 7.22 (d, 2H, Ar-H), 6.33 (t, 1H, -CH), 3.31 (s, 2H, -CH), 2.62 (t, 2H, -CH), 2.54 (s, 3H, -CH), 2.40 (m, 2H, -CH_2_). IR (KBr, cm^-1^): 3018, 2928, 2535, 1646, 718. MS (ESI) *m/z*: 208.9(M+H^+^). Anal. Calc. for C_12_H_14_ClN (in %): C-69.39, H-6.79, N-6.74. Found C-69.35, H-6.76, N-6.70.

#### 4.3.5 Synthesis of 3-(4-methoxyphenyl)-1,2,5,6-tetrahydro-1-methylpyridine 5e

Obtained from 3-(4-methoxy-phenyl)-1-methyl-pyridinium salt (**4e**), (0.5 g, 1.52 mmol), NaBH_4_ (0.28 g, 7.64 mmol). The product obtained was oily liquid (0.279 g, 90%). 1,2,3, 6-tetrahydro-5-(4-methoxyphenyl)-1-methylpyridine. ^1^ H-NMR (CDCl_3_, 400MHz) δ: 7.25 (d, 2H, Ar-H), 7.00 (d, 2H, Ar-H), 6.24 (t, 1H, -CH), 3.83 (s, 3H, -OCH_3_), 3.33 (s, 2H, -CH), 2.63 (t, 2H, -CH), 2.60 (s, 3H, -CH), 2.44 (m, 2H, -CH). IR (KBr, cm^-1^): 3012, 2931, 2512, 1643, 1193. MS (ESI) *m/z*: 204.3(M+H^+^). Anal. Calc. for C_13_H_17_NO (in %): C-76.81, H-8.43, N-6.89. Found C-76.78, H-8.39, N-6.85.

#### 4.3.6 Synthesis of 3-(2,6-dichlorophenyl)-1,2,5,6-tetrahydro- 1-methylpyridine 5f

Obtained from 3-(2,6-dichloro-phenyl)-1-methyl-pyridiniumsalt (**4f**), (0.5 g, 1.36 mmol), NaBH_4_ (0.258 g, 6.82 mmol). The product obtained was oily liquid (0.273 g, 83%).^1^H NMR (CDCl_3_, 400MHz) δ: 7.64 (d, 2H, Ar-H) 7.52 (t, 1H, Ar-H), 6.26 (t, 1H, -CH), 3.4 (s, 2H, -CH), 2.66 (t, 2H, -CH), 2.54 (s, 3H, -CH), 2.44 (m, 2H, -CH). IR (KBr, cm^-1^): 3027, 2965, 2532, 1622, 707. MS (ESI) *m/z*: 242.1(M+H^+^). Anal. Calc. for C_12_H_13_Cl_2_N (in %): C-59.92, H-5.41, N-5.78. Found C-59.89, H-5.38, N-5.75.

#### 4.3.7 Synthesis of 3-(4-phenoxyphenyl)-1,2,5,6-tetrahydro-1-methylpyridine 5g

Obtained from 3-(4-phenoxyphenyl)-1-methyl-pyridinium salt (**4g**), (0.5 g, 1.28 mmol), NaBH_4_ (0.242 g, 6.42 mmol). The product obtained was oily liquid (0.289 g, 85%). ^1^H NMR (CDCl_3_, 400MHz) δ: 7.45 (d, 4H, Ar-H), 7.25-7.35 (m, 5H, Ar-H), 6.28 (t, 1H, -CH), 3.35 (s, 2H, -CH), 2.62 (t, 2H, -CH), 2.52 (s, 3H, -CH), 2.43 (m, 2H, -CH). IR (KBr, cm^-1^): 3126, 2943, 2528, 1642, 1370. MS (ESI) *m/z*: 266.10(M+H^+^). Anal. Calc. for C_18_H_19_NO (in %): C-81.47, H-7.22, N-5.28. Found C-81.44, H-7.18, N-5.25.

#### 4.3.8 Synthesis of 3-(2-fluoro-biphenyl-4-yl)-1-methyl-1,2,5,6-tetrahydropyridine 5h

Obtained from 3-(2-fluoro-biphenyl-4-yl)-1-methyl-pyridinium salt (**4h**), (0.5 g, 1.27mmol), NaBH_4_ (0.241 g, 6.39 mmol). The product obtained was oily liquid (0.303 g, 89%). ^1^H NMR (CDCl_3,_ 400MHz) δ: 7.7 (d, 1H, Ar-H), 7.40 (d, 2H, Ar-H), 7.25-7.35 (m, 5H, Ar-H), 6.34 (t, 1H, -CH), 3.24 (s, 2H, -CH), 2.61 (t, 2H, -CH), 2.52 (s, 3H, -CH), 2.41 (m, 2H, -CH). IR (KBr, cm^-1^): 3140, 2934, 2538, 1634, 1228. MS (ESI) *m/z*: 268.20(M+H^+).^ Anal. Calcd. for C_18_H_18_FN (in %): C-80.87, H-6.79, N-5.24. Found C-80.84, H-6.75, N-5.22.

#### 4.3.9 Synthesis of 3-(phenol-3-yl) 1,2,5,6-tetrahydro-1-methylpyridine 5i

Obtained from 3-(phenol-3-yl) -1-methyl-pyridinium salt (**4i**), (0.5 g, 1.60 mmol), NaBH_4_ (0.302 g, 8.0 mmol). The product obtained was oily liquid (0.259 g, 86%). ^1^H NMR (CDCl_3_, 400MHz) δ: 7.4 (s, 1H, Ar-H), 6.92-7.25 (m, 3H, Ar-H), 6.28 (t, 1H, -CH), 3.22 (s, 2H, -CH), 2.58 (t, 2H, -CH), 2.49 (s, 3H, -CH), 2.44 (m, 2H, -CH). IR (KBr, cm^-1^): 3610, 3148, 2943, 2531, 1627. MS (ESI) *m/z*: 190.08(M+H^+^). Anal. Calc. for C_12_H_15_NO (in %): C-79.16, H-7.99, N-7.40. Found C-79.95, H-7.96, N-7.37.

#### 4.3.10 Synthesis of 3-(3-ethoxyphenyl)-1,2,5,6-tetrahydro-1-methylpyridine 5j

Obtained from 3-(3-ethoxyphenyl) pyridine salt (**4j**), (0.5 g, 1.46 mmol), NaBH_4_ (0.277 g, 7.30 mmol). The product obtained was oily liquid (0.260 g, 82%). ^1^H NMR (CDCl_3_, 400MHz) δ: 7.38 (s, 1H, Ar-H), 6.89-7.28 (m, 3H, Ar-H), 6.24 (t, 1H, C-H), 3.5 (q, 2H, -CH_2_), 3.22 (s, 2H, -CH), 2.58 (t, 2H, -CH), 2.52 (s, 3H, -CH_3_), 2.44 (m, 2H, -CH), 1.62 (t, 3H, -CH_3_). IR (KBr, cm^-1^): 3146, 2965, 2523, 1633. MS (ESI) *m/z*: 218.10(M+H^+^). Anal. Calc. for C_14_H_19_NO (in %): C-77.38, H-8.81, N-6.45. Found C-77.35, H-8.78, N-6.42.

#### 4.3.11 Synthesis of 3-(3-chloro-2-fluoro-phenyl)-1-methyl-1,2,5,6-tetrahydropyridine 5k

Obtained from 3-(3-Chloro-2-fluoro-phenyl)-pyridinum salt (**4k**), (0.5 g, 1.43 mmol), NaBH_4_ (0.270 g, 7.32 mmol). The product obtained was oily liquid (0.0.268 g, 83%). ^1^H NMR (CDCl_3_, 400MHz) δ: 7.45 (d, 1H, Ar-H), 7.27 (d, 1H, Ar-H), 7.12 (t, 1H, Ar-H), 6.29 (t, 1H, -CH), 3.25 (s, 2H, -CH_2_), 2.63 (t, 2H, -CH_2_), 2.58 (s, 3H, -CH_3_), 2.51 (m, 2H, -CH_2_). IR (KBr, cm^-1^): 3156, 2536, 1632, 1232, 760. MS (ESI) *m/z*: 227.08(M+H^+^). Anal. Calc.for C_12_H_13_ClFN (in %): C-63.86, H-5.81, N-6.21. Found C-63.85, H-5.79, N-6.19.

### 4.4 Biology: *In Vitro* Cholinesterase Assay

The cholinesterase assay was done by using the method described by Ellman *et al.* [30] to determine the *in vitro* cholinesterase activity. The activity was measured by the increase in absorbance at 412nm due to the yellow color produced from the reaction of thiocholine with the dithiobisnitrobenzoate ion. Rat brain AChE was obtained from the brain of wistar rat by homogenizing under Teflon blender for 10 minutes in 0.1M KH_2_PO_4_ buffer pH 8. A stock solution of Enzyme in 0.1M KH_2_PO_4_ buffer (pH 8.0) was kept frozen. For each assay, 300μg of enzyme was used; acetylthiocholine iodide was prepared daily using 0.1M KH_2_PO_4_ buffer (pH 7.0). A 0.01M solution of DTNB was prepared in 0.1M KH_2_PO_4_ buffer (pH 7.0). Crude human AChE was obtained by mixing 9 ml of fresh blood (collected from healthy volunteer by vein puncture) with 1ml of 3.8 % (w/v) trisodium citrate and centrifuging at 3000rpm at 0°C for 20min. The supernatant was used as a source of AChE. Electric eel AChE was obtained from sigma laboratory and similar procedure was employed for the assay as that of rat brain AChE.

## Experimental Condition and Kinetics

Enzyme activity was measured using Shimadzu Spectrophotometer. The assay medium contained phosphate buffer, pH 8.0 (2.6 ml), DTNB (0.1 ml), 5 μl of enzyme, 20 μ l of 0.075 M substrate. The activity was determined by measuring the increase in absorbance at 412 nm at 1 minute interval for 10 minutes at 37°C. In dose dependent inhibition studies, the substrate was added to the assay medium containing enzyme, buffer and DTNB with inhibitor after 10 minutes of incubation time. Calculations were performed according to the method of the equation in Ellman *et al*. All experiments were carried out in triplicate and the mean values are reported here. The relative activity was expressed as percentage ratio of enzyme activity in the absence of inhibitor.

### Protein Estimation

Protein content was determined by Lowry method [31] using bovine serum albumin as standard.

### IC_50_ Determination

AChE inhibitor Neostigmine (a reversible cholinesterase inhibitor), was used in the concentration range 10 to 90 nM to inhibit the AChE of electric eel, human serum, and rat brain homogenate [32]. Inhibition by tetrahydro pyridine derivatives was studied in the presence of different concentrations of compounds and the percentage inhibition of enzyme activity was calculated. The inhibition of AChE by tetrahydro pyridine derivatives was analyzed with values obtained in comparison to that of Neostigmine. Antiamnesic effect was carried out for synthesized tetrahydro pyridine derivatives against scopolamine induced memory loss using passive avoidance step-down task paradigm in rats according to the method of Sharma and Kulkarni [33, 34].

## Figures and Tables

**Fig. (1):**
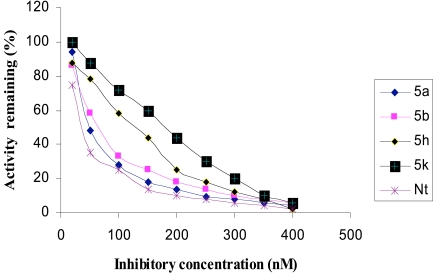
Inhibition of electric eel AChE by tetrahydropyridine derivatives.

**Fig. (2):**
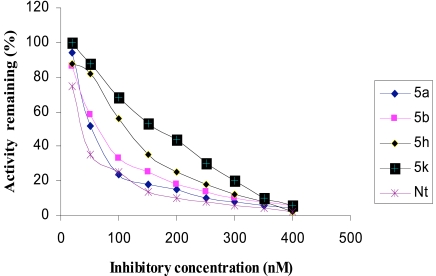
Inhibition of human serum AChE by tetrahydropyridine derivatives.

**Fig. (3):**
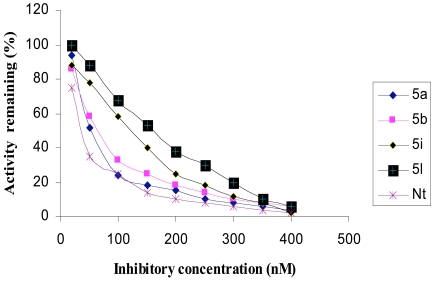
Inhibition of rat brain homogenate AChE by tetrahydropyridine derivatives.

**Fig. (4):**
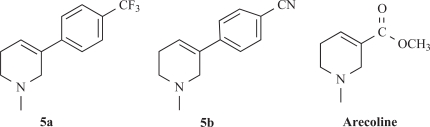
Structures of potent AChE inhibitors and Arecoline.

**Table 1:** Chemical Structure, Yield and Purity of the Synthesized Compounds 5a-k

Compound	R	Yield (%)	Purity (%)
**5a**	4-CF_3_	85	97.1
**5b**	4-CN	80	96.4
**5c**	3-COCH_3_	77	98.5
**5d**	4-Cl	88	96.3
**5e**	4-OCH_3_	90	98.2
**5f**	2,6-dichloro	83	99.0
**5g**	4-C_6_H_5_O	85	96.4
**5h**	2-F-4-phenyl	89	97.5
**5i**	3-OH	86	98.5
**5j**	3-OC_2_H_5_	82	97.4
**5k**	2-F, 3-Cl	83	98.3

**Table 2:** Comparative Inhibitory Activities Shown by the Tetrahydropyridine Derivatives 5a-k Against AChE from Different Sources

**Compound**	Electric eel AChE, IC_50_ nM	Human Serum AChE, IC_50_ nM	Rat brain Homogenate AChE, IC_50_ nM
**5a**	**49.5**	**52**	**51.6**
**5b**	**64.0**	**68.0**	**63.6**
**5c**	NI	NI	NI
**5d**	806	784	827
**5e**	NI	2876	NI
**5f**	678	729	704
**5g**	1234.8	1350.4	1289.2
**5h**	118	115.6	122.6
**5i**	NI	NI	NI
**5j**	1234.1	1224.6	1238
**5k**	182.3	170	176.8
**Neostigmine**	36.5	39.2	38.4

Values are means of three determinations, the ranges of which were less than 5% of the mean in all cases.

NI, no inhibition found.

**Table 3:** Study of Antiamnesic Effect of Tetrahydropyridine Derivatives Against Scopolamine Induced Memory Loss

S. No.	Experimental Groups	Treatment (Dose) mg/kg ip	Basal Latecy (s) of Rat to Reach Shock- Free Zone (SFZ)			Memory Parameters	
			I	II	III	Latency (s)	No. of Mistakes
1	Control groups	-	19	4	0.9	1	8
2	Scopolamine treated	0.4	35	10	8	4	34
3	**5a treated groups**	0.1	22	8	4	3	13
4	**5b**	0.1	25	9	5	4	15
5	**5c**	0.1	34	9	8	4	32
6	**5d**	0.1	30	8	7	4	30
7	**5e**	0.1	33	9	8	4	33
8	**5f**	0.1	30	8	6	3	30
9	**5g**	0.1	30	8	7	4	30
10	**5h**	0.1	26	6	3	3	18
11	**5i**	0.1	34	9	8	4	33
12	**5j**	0.1	30	8	7	4	30
13	**5k**	0.1	28	6	5	3	20

## References

[1] Cummings JL, Askin-Edgar S (2000). Evidence for Psychotropic Effects of Acetylcholinesterase Inhibitors. CNS Drugs.

[2] Cutler NR, Sramek JJ (2001). Review of the next generation of Alzheimer's disease therapeutics: challenges for drug development. Prog. Neuro-Psychopharmacol. Biol. Psychiat.

[3] Gauthier S (2001). Alzheimer's disease: current and future therapeutic perspectives. Prog. Neuro-Psychopharmacol. Biol. Psychiat.

[4] Kumar V, Sugaya K, Saunders S, Mechanic J (1996). Update on cholinergic drugs in Alzheimer’s Disease. Drugs Today.

[5] Lander CJ, Lee JM (1998). Pharmacological Drug Treatment of Alzheimer Disease: The Cholinergic Hypothesis Revisited. J. Neuropathol. Exp. Neurol.

[6] Hollander E, Mohs RC, Davis KL (1986). Cholinergic approaches to the treatment of Alzheimer's disease. Br. Med. Bull.

[7] Hermann C, Stern RG, Losonzcy MF, Jaff S, Davidson M (1991). Diagnostic and pharmacolocigal approaches in Alzheimer's disease. Drugs Aging.

[8] Eagger SA, Levy R, Sahakian BJ (1991). Tacrine in Alzheimer's disease. Lancet.

[9] Lamy PP (1994). The role of cholinesterase inhibitors in Alzheimer's disease. CNS Drugs.

[10] Maltby N, Broe GA, Creasey H, Jhnn AF (1994). Efficacy of tacrine and lecithin in mild to moderate Alzheimer's disease: double blind trial. BMJ.

[11] Brodaty H (1999). Realistic expectations for the management of Alzheimer's disease. Eur Neuropsychopharmacol.

[12] Schachter AS (1999). Guidelines for the Appropriate Use of Cholinesterase Inhibitors in Patients with Alzheimer's Disease. CNS Drugs.

[13] Mohs RC, Davis BM, Johns CA, Mathe AA, Greenwald BS, Horvath TB (1985). Oral physostigmine treatment of patients with Alzheimer's disease. Am J Psychiatry.

[14] Schneider LS, Tariot PN (1994). Emerging drugs for Alzheimer's disease. Mechanisms of action and prospects for cognitive enhancing medications. Med Clin North Am.

[15] Thal LJ, Fuld PA, Masur DM, Sharpless NS (1983). Oral physostigmine and lecithin improve memory in Alzheimer disease. Ann Neurol.

[16] Farlow M, Grascon SI, Hershey LA, Lewis KW, Sadowsky CH, Dolan-ureno J (1992). A controlled trial of tacrine in Alzheimer's disease. J Am Med Assoc.

[17] Dinnendahl V, Stock K (1975). Effects of arecoline and cholinesterase inhibitors on cyclic guanosine 3',5'-monophosphate and adenosine 3'.5'-monophosphate in mouse brain. Naunyn-Schmiedebergs Arch Pharmacol.

[18] Galliani G, Cesana R, Barzaghi F (1987). Reversal of scopolamine-induced amnesia by acrecoline. Med Sci Res.

[19] Ridley RM, Baker HF, Drewett B (1987). Effects of arecoline and pilocarpine on learning ability in marmosets pretreated with hemicholinium-3. Psycho-pharmacol.

[20] Christie JE, Shering A, Ferguson J, Gleu AJM (1981). Physostigmine and arecoline: effects of intravenous infusions in Alzheimer presenile dementia. Br J Psychiatr.

[21] Sitaram N, Weingartner H, Gillin JC (1978). Human serial learning: enhancement with arecholine and choline impairment with scopolamine. Science.

[22] Holmstedt B, Lundgren G (1967). Arecoline, nicotine, and related compounds: tremorgenic activity and effect upon brain acetylcholine. Ann NY Acad Sci.

[23] Nieschulz O, Schmersahl P (1968). On the pharmacology of active materials from betel-2 Transformation of arecoline to arecaidine. Arzneimittelforschung.

[24] Gernard Vogel H, Wolfgang Vogel (1997). Drug Discovery and Evaluation: Pharmacological Assays.

[25] Kato K, Hayako H, Ishihara Y, Marui S, Iwane M, Miyamoto M (1999). TAK-147, an acetylcholinesterase inhibitor, increases choline acetyltransferase activity in cultured rat septal cholinergic neurons. Neurosci Lett.

[26] Kameyama T, Nabeshima T, Noda T (1986). Cholinergic modulation of memory for step-down type passive avoidance task in mice. Research communications in psychology, psychiatry and behavior.

[27] Bartus RT, Dean RL, Beer B, Lippa AS (1982). The cholinergic hypothesis of geriatric memory dysfunction. Science.

[28] Jin-Heng Li, Xi-Chao Hu, Ye-Xiang Xie (2006). Polymer-supported DABCO-palladium complex as a stable and reusable catalyst for room temperature Suzuki-Miyaura cross-couplings of aryl bromides. Teterahedron Lett.

[29] Sunil Kumar YC, Sadashiva MP, Rangappa KS (2007). An Efficient Synthesis of 2-(1-methyl-1,2,5,6-tetrahydropyridine-3-yl)morpholine: a potent M_1_ selective muscarinic agonist. Teterahedron Lett.

[30] Ellman GL, Courtneykd, Andress V, Eartherstone FM (1961). A new and rapid colorimetric determination of acetylcholinesterase activity. Biochem Pharmacol.

[31] Lowry OH, Rosenbrough NJ, Farr AL, Randall RJ (1951). Protein measurement with the Folin phenol reagent. J Biol Chem.

[32] Ohtaka, Kanazawa T, Ito K, Tsukamoto G (1987). Benzylpiperazine derivatives. IV Syntheses and cerebral vasodilating activities of 1-benzyl-4-diphenylmethylpiperazine derivatives. Chem Pharm Bull.

[33] Sharma AC, Kulkarni SK (1990). Evidence for GABA-BZ receptor modulation in short-term memory passive avoidance task paradigm in mice. Methods Find. Exp. Clin. Pharmacol.

[34] Sharma AC, Kulkarni SK (1991). Effects of MK-801 and ketamine on short-term memory deficits in passive avoidance step-down task paradigm in mice. Methods Find. Exp. Clin. Pharmacol.

